# Analysis of the Accuracy and Inter-Reader Precision of Scar Quantification Techniques in Aortic Stenosis: A Comparative Cardiovascular Magnetic Resonance Imaging Study

**DOI:** 10.3390/diagnostics16132017

**Published:** 2026-06-28

**Authors:** Megan Rian Rajah, Pieter-Paul Strauss Robbertse, Vishesh Sood, Tonya Marianne Esterhuizen, Anton Frans Doubell, Philip George Herbst

**Affiliations:** 1 Division of Cardiology, Department of Medicine, Faculty of Medicine and Health Sciences, Stellenbosch University, Tygerberg Hospital, Cape Town 7505, South Africa; 2 Department of Radiology, SCP Radiology, Panorama Mediclinic Hospital, Cape Town 7500, South Africa; 3 Division of Epidemiology and Biostatistics, Department of Global Health, Stellenbosch University, Cape Town 7505, South Africa

**Keywords:** myocardial fibrosis, fibrosis quantification, late gadolinium enhancement, aortic stenosis, cardiovascular magnetic resonance imaging, aortic valve replacement

## Abstract

**Background:** An important determinant of mortality in AS is the presence and quantity of myocardial scar. Scar quantification using late gadolinium enhancement (LGE) on cardiovascular magnetic resonance (CMR) imaging may be a useful risk-stratification tool for at-risk patients who do not meet current criteria for valve intervention. The incorporation of this tool into clinical practice is currently limited by a lack of consensus on the best LGE quantification technique to use. **Methods:** Fifteen patients with severe AS underwent LGE imaging on CMR. A reference estimate of the LGE mass was made using a semi-automatic quantitative visual method. An intensity slider (reporting software provided) was used to mark areas of enhanced signal in each short-axis slice that correlated with the reader’s visual assessment of LGE, which used predetermined imaging criteria. This visual slider method (VslM) of determining LGE mass was then used as a reference for establishing the accuracy of various semi- and fully automated methods for identifying and quantifying LGE burden. These included the signal threshold versus reference mean (STRM) method at thresholds of two-, three-, and five-standard deviations (2SD, 3SD, 5SD, respectively), the full width at half maximum (FWHM) method and the Otsu auto threshold (OAT) method. An intraclass correlation analysis was performed to establish and compare the inter-reader reliability for each method. **Results:** Three readers demonstrated 100% agreement on the presence of LGE in 12/15 (80%) of study cases. Accuracy determined by the Wilcoxon rank sum, Spearman correlation and Bland–Altman tests suggested that the 5SD method using remote myocardium reference regions of interest only in slices with visually detected LGE was best (Wilcoxon rank sum *p*-values ranged from 0.3 to 0.5 for the three readers, bias on Bland–Altman was <0.5 g for all three readers). This was followed by the FWHM method, but with wide minimum-maximum ranges observed. Inter-reader reliability was best for the 2SD STRM method (ICC = 0.9, *p* < 0.001), but accuracy using this method was clinically unacceptable. Inter-reader reliability was statistically acceptable for the VslM (ICC = 0.7, *p* < 0.001). The FWHM method yielded the best balance between accuracy and reliability but may be limited by the heterogeneity of scars observed from patient to patient. **Conclusions:** The FWHM appeared to offer a reasonable balance between accuracy and precision. However, it was not always the best fit, e.g., in patients with small or non-bright scars. There may be no additional benefit to using the semi- and fully automated methods in the context of AS, and visual estimation, when performed in the manner described in this study (i.e., the VslM), may be clinically sufficient.

## 1. Introduction

Aortic stenosis (AS) is one of the most common valvular lesions with a growing prevalence worldwide [1]. Definitive treatment by means of surgical aortic valve replacement (SAVR) or transcatheter aortic valve implantation (TAVI) is indicated in those with severe disease and symptoms or severe disease with systolic dysfunction (left ventricular ejection fraction < 50%) [2,3,4]. Despite the widespread implementation of these interventions, the morbidity and mortality remain high [1]. Furthermore, a mortality risk in groups not meeting current guideline recommendations for intervention has also been shown [2,3,5,6]. This raises two important questions: firstly, whether the current guideline recommendations for the timing of intervention can be improved; and secondly, whether the presence/absence of other high-risk clinical parameters warrants their incorporation into a new risk stratification strategy for reclassifying high-risk patients not qualifying for valvular intervention. These parameters include evidence of myocardial injury using biomarkers such as brain natriuretic peptide/amino-terminal B-type natriuretic peptide (BNP/NT-pro BNP, respectively), abnormal strain analysis on speckle-tracking echocardiography, and the presence and quantity of myocardial fibrosis [2,3].

Myocardial fibrosis in AS is well-described on histology and cardiovascular magnetic resonance (CMR) imaging, and it takes on two distinct patterns—diffuse interstitial fibrosis and replacement fibrosis/scar [7,8]. Replacement fibrosis/scar in AS is associated with an increased mortality risk, and this relationship has been shown to be dose-dependent [9,10,11,12,13,14,15,16]. Cardiovascular magnetic resonance imaging offers clinicians a validated approach for the assessment of replacement fibrosis through late gadolinium enhancement (LGE) imaging, which circumvents the invasive nature, required expertise, and issues of sampling bias associated with endomyocardial biopsy/histology [17,18,19,20]. The detection of LGE requires the use of inversion-recovery magnetic resonance sequences planned specifically to null the background/normal myocardium, against which hyperenhanced areas where gadolinium is concentrated can be visually assessed [21,22,23,24]. This visual identification of bright LGE areas remains the clinical mainstay for the detection of replacement fibrosis on CMR [24]. Several post-processing methods for the quantification of LGE exist. However, consensus as to which method is best for replacement fibrosis quantification in AS has yet to be reached, thus limiting its adoption into clinical practice [24,25].

The methods used for LGE quantification in AS include a visual method and a variety of semi-automated ones [24,25,26]. As described, the mainstay of LGE detection on CMR relies on the visual identification of bright/hyperenhanced myocardial areas. This visual assessment has been further developed and used as a robust but rather blunt quantitative instrument, requiring the user to broadly classify the transmural proportion of LGE per left ventricular (LV) segment (typically, 0–25%, 25–50%, 50–75% and 75–100%) as representing the proportional extent of replacement fibrosis in those segments [24]. By tallying the proportions of each individual segment affected by replacement fibrosis, a global LV scar burden may be estimated. Although demonstrated to be robust, with data in a number of different pathologies [24,27,28,29], this is a time-consuming endeavour and results in a broad categorisation in terms of the scar burden. A number of semi- and fully automated methods have been developed in an attempt to refine the visual method of replacement fibrosis by narrowing the broad categories to more specific estimates that better approximate the visual assessment of the observer. In addition to improving the accuracy of the traditional visual method, these approaches also aim to improve the speed and efficiency of LGE assessment, as well as the reproducibility of its quantification [25]. Some methods were specifically developed to reduce bias by introducing a user-selected reference region (remote myocardial reference regions representing normal myocardium or bright LGE reference regions representing scar, depending on the methodology used) that defines a slice-by-slice signal-intensity threshold above which pixels are automatically identified as LGE and quantified as such.

Several challenges with the use of these semi-automated methods in AS have previously been described in detail by our group [25] and include the persistence of bias and poor reproducibility due to a critical dependence on the size and positioning of the user-defined reference regions, as well as the coexistence of diffusely distributed interstitial fibrosis. The presence of relatively small, non-bright scars drives further complexity in interpreting the results. An interesting semi-quantitative visual method, termed the visual slider method (VslM), allows the reporting physician to manually adjust a signal-intensity threshold slider, above which pixels are highlighted and counted. The operator can therefore visually adjust the slider until relevant areas identified as true LGE are marked and counted as fibrosis. This allows the operator to manually curate areas deemed fibrosis while narrowing the scar quantity categorisation compared to the broad output of the traditional visual method, and while avoiding the challenges associated with the semi-automated methods.

A head-to-head analysis of the various LGE quantification methods in AS remains limited. A single study of this nature exists where a range of standard deviation (SD) thresholds within the signal threshold versus reference mean (STRM) method was evaluated [8]. A head-to-head analysis that includes an operator-curated visual method, such as the VslM, has not been studied, and alternatives to the STRM, such as the full width at half maximum (FWHM) and Otsu auto threshold (OAT) methods, have yet to be reported for AS. This study aimed to compare the accuracy and precision/inter-reader reproducibility of five semi-automated LGE quantification methods using an operator-curated, semi-automated estimate of replacement fibrosis, the VslM, as the reference standard. The study also aimed to illustrate the impact of different post-processing styles on the accuracy and precision of the various LGE quantification methods.

## 2. Methods

### 2.1. Study Population

The study population comprised fifteen participants selected from a larger study at Tygerberg Hospital, Cape Town, South Africa. All participants met the guideline criteria for high-gradient severe AS (aortic valve area < 1.0 cm^2^, mean transvalvular pressure gradient ≥ 40 mmHg, peak velocity ≥ 4 m/s) with and without systolic dysfunction as diagnosed on transthoracic echocardiography [2,3]. Patients with other haemodynamically significant valve lesions, concomitant cardiomyopathy including cardiac amyloidosis, previous myocardial infarction and/or significant coronary artery disease were excluded from the study. Significant coronary artery disease (CAD) was determined by coronary angiography (obstruction of >50% was regarded as significant). The study obtained ethical approval from the Human Research Ethics Committee of Stellenbosch University (S21/11/251 PHD), and written informed consent was obtained from all participants.

### 2.2. Image Acquisition

Cardiovascular magnetic resonance imaging was performed using a Magnetom Aera (Siemens, Erlangen, Germany) 1.5 Tesla scanner. A standardised protocol in accordance with the Society of Cardiovascular Magnetic Resonance’s recommendations was used [23]. Late gadolinium enhancement images were acquired 10–15 min after contrast injection (Gadovist 0.2 mmol/kg, Bayer Pharmaceuticals, Leverkusen, Germany). An electrocardiogram-gated, breath-held, inversion-recovery gradient-echo sequence was used for the image acquisition. Images were acquired in the standard cardiac planes, which included two-, three-, and four-chamber long-axis views and a short-axis stack covering from the base to the apex of the LV. The choice of inversion time (TI) was selected using a modified look-locker inversion-recovery TI scout. Typical image parameters included an 8 mm slice thickness with no gap where applicable, and TE. 1.20–1.89 ms, TR. 784–804 ms, TI 260–280 ms, and matrix size 208 × 256 with voxel size ± 1.4 × 0.5 × 8.0 mm.

### 2.3. Image Post-Processing

The LGE images of all fifteen participants were independently post-processed by three CMR readers with six (PPRS), four (MRR) and four (VS) years of CMR experience, respectively. Two of the readers were blinded to the clinical data, to other imaging modalities, and to the LGE measurements of the other readers. Access to the cine images was available to guide the endo- and epicardial contouring. Other CMR measurements, e.g., LV volumes and parametric mapping times, however, were not made available to the two blinded readers. One reader served as the primary investigator for the larger cohort and was therefore not blinded to the clinical information or other imaging modalities and measurements.

The images were post-processed using commercially available software (CVI42, version 5.13.7, Circle Cardiovascular Imaging, Calgary,Canada). A visual analysis was initially performed to identify areas of hyperenhancement in accordance with standard practice [24]. Areas of enhancement were diagnosed if visualised in two orthogonal planes and on a phase swap of the original image. Following a suitable level of windowing, endo- and epicardial borders were contoured on every slice of the short-axis LGE stack. The VslM was chosen as a reference method and is described in further detail below. Semi-automated methods tested against the reference included (1) the signal threshold versus reference mean method (STRM) at thresholds of two-, three-, and five-standard deviations (2SD, 3SD, 5SD, respectively) above the mean signal intensity of remote myocardium, (2) the full-width at half maximum (FWHM) method and (3) the Otsu auto-threshold (OAT) method. For the STRM method, a normal myocardial region of interest (ROI) was initially drawn only in those slices identified by the reader as containing LGE (denoted by the letter “A” in the text) and then in every slice of the short-axis stack irrespective of whether LGE was identified by the visual assessment of the CMR reader (denoted by the letter “B” in the text). For method A, the extrapolation setting (a setting that applies the mean signal intensity of the ROI to the ROI-free adjacent slices) was turned off. Therefore, those slices deemed LGE-free by the CMR reader were excluded from the analysis without impacting the total myocardial volume used for the total LGE proportion (%) calculation. Care was taken to avoid the endo- and epicardial borders when drawing the myocardial ROI. For the FWHM method, an enhancement ROI was drawn in the core of the scar to minimise the impact of partial volume effects arising from the scar edges. As for the STRM method, two post-processing techniques were compared: a single ROI was drawn with extrapolation on (denoted by the letter “A” in the text), and ROIs in every scar present with extrapolation on were also drawn (denoted by the letter “B” in the text). The LGE mass (grams) and proportion (%) of total myocardial volume were recorded for analysis.

### 2.4. Reference Method

**Figure 1:**
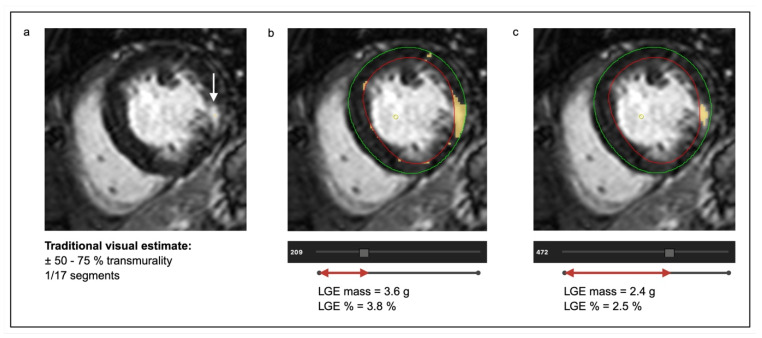
Illustration of the visual slider method (VslM) of LGE quantification. (**a**) Inferolateral subendocardial LGE is shown by the white arrow. The traditional visual assessment yields a transmural extent of ±50–75% in one of seventeen LV segments. (**b**,**c**) The semi-quantitative VslM approach. The yellow-shaded region illustrates the pixels counted as enhanced, based on the manual adjustment of a slider function/tool (depicted below each short-axis image) by the CMR reader. This function is available on CVI42 software, which provides a cumulative, quantitative output in the form of LGE mass (grams) and proportion LGE (%) for each slice analysed. VslM—visual slider method, LGE—late gadolinium enhancement, LV—left ventricular.

A visual slider method (denoted by the abbreviation “VslM”) was selected as a reference against which other methods were compared. Rather than the traditional visual method (Figure 1a) of user-estimated transmural extent/proportional area LGE (%) per LV segment, a semi-quantitative approach was employed using the “manual per slice” option offered by CVI42 (Figure 1b,c). Endo- and epicardial borders were contoured on every slice of the short-axis stack. A slider function, guided by the reader’s visual assessment, was then manually adjusted (slice-by-slice) to mark pixels considered as hyperenhanced. This yielded an objective estimate of the LGE mass (grams) and the proportion of LGE (%) of total myocardial volume. This pixel-wise quantification enabled a more objective visual estimate than the traditional visual approach (Figure 1).

### 2.5. Statistical Analysis

Statistical analysis was performed using GraphPad Prism (version 10.0.2, GraphPad, Boston, TX, USA) and SPSS (version 29.0.2.0, IBM, New York, NY, USA). Normality tests were performed using the Shapiro–Wilk test, histograms, and quantile-quantile plots. Accuracy was tested using paired Wilcoxon rank-sum tests, Spearman’s correlation (reported as rho with a 95% confidence interval and *p* value) and Bland–Altman analyses (reported as bias ± standard deviation). For the Bland–Altman analysis, a natural log transformation was performed, and the normality of the differences was confirmed using the Shapiro–Wilk test. For the inter-reader reliability evaluation, intraclass correlation (ICC) analysis was performed on the normalised data. The images of all fifteen participants were read by the same three, non-specific readers. A two-way random effects model was therefore selected [30]. The ICC for the absolute agreement of single measurements between readers for each method was reported together with its *p*-value.

## 3. Results

### 3.1. Study Population

The baseline characteristics of the study population are shown in Table 1. Fifteen patients with high-gradient severe AS were analysed for this study. The mean aortic valve area was 0.51 ± 0.15 cm^2^ with a mean gradient of 61 ± 18 mmHg. Patients with and without systolic dysfunction were included, with a mean LV ejection fraction of 47 ± 18%. The prevalence of hypertension was high [13 (87%)], but with well-controlled blood pressure readings (systolic blood pressure 126 ± 22 mmHg and diastolic blood pressure 73 ± 12 mmHg). There were no cases of CAD (determined by coronary angiography) or cardiac amyloidosis (determined by CMR).

### 3.2. Accuracy by Method Used

**Figure 2:**
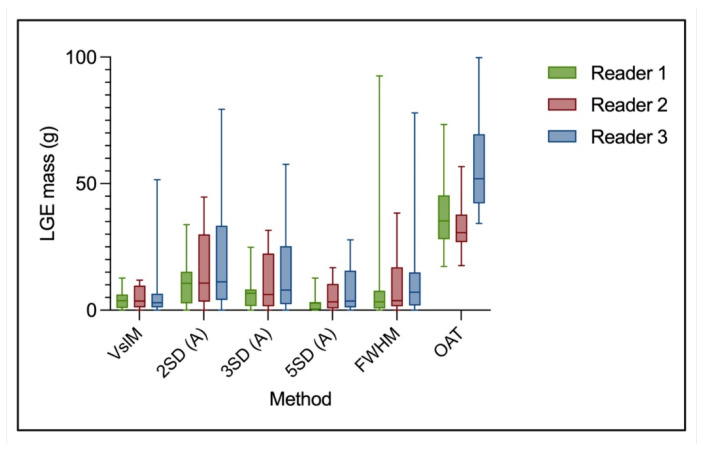
Column graph displaying the median and interquartile range of LGE mass determined using six different methods amongst three different readers. The median LGE mass, when compared to that of the VslM, varied significantly for the 2SD (A), 3SD (A) and OAT methods amongst all three readers. The 5SD (A) method for readers two and three and the FWHM method for readers one and two yielded non-significant differences in the median LGE mass when compared with the VslM. Significance was determined using the Wilcoxon rank-sum test, and a *p*-value < 0.05 was considered significant. LGE—late gadolinium enhancement, VslM—visual slider method, 2SD (A)—two standard deviations method A, 3SD (A)—three standard deviations method A, 5SD (A)—five standard deviations method A, FWHM—full width at half maximum, OAT—Otsu auto threshold.

Of fifteen severe AS cases, three were found to have no LGE (determined by visual assessment) with 100% agreement amongst the three readers. As shown in Figure 2, the median LGE mass varied significantly according to the method used. Compared to the reference method (VslM), the largest absolute differences were obtained using the OAT method [reader one: 3.8 (0.9–6.2 g) vs. 35.5 (28.0–45.4 g), reader two: 3.6 (1.2–9.7 g) vs. 30.6 (26.9–37.8 g) and reader three: 3.0 (1.1–6.5 g) vs. 52.0 (42.2–69.6 g) respectively]. Using paired Wilcoxon rank-sum tests and the VslM as a reference, significant differences (*p* > 0.05) were found for the 2SD (A), 3SD (A) and OAT methods across all three readers (Supplementary Table S1). No statistically significant differences were found for readers two and three when using the 5SD (A) method and for readers one and two when using the FWHM (A) method (Supplementary Table S1). Significant, near-perfect linear relationships were found for all methods when compared to the VslM except for the OAT method (Spearman’s rho ranged from 0.1 to 0.4 across readers) (Supplementary Table S1). The smallest bias was observed using the 5SD (A) method, and this finding was consistent across all three readers (reader one: −0.03 ± 1.37, reader two: 0.03 ± 0.76 and reader three: 0.32 ± 0.69) (Supplementary Table S2). The largest bias was observed using the OAT method (reader one: 2.81 ± 1.60, reader two: 2.20 ± 0.94 and reader three: 2.77 ± 1.08) (Supplementary Table S2).

### 3.3. Accuracy and Precision by Post-Processing Method Used

**Figure 3:**
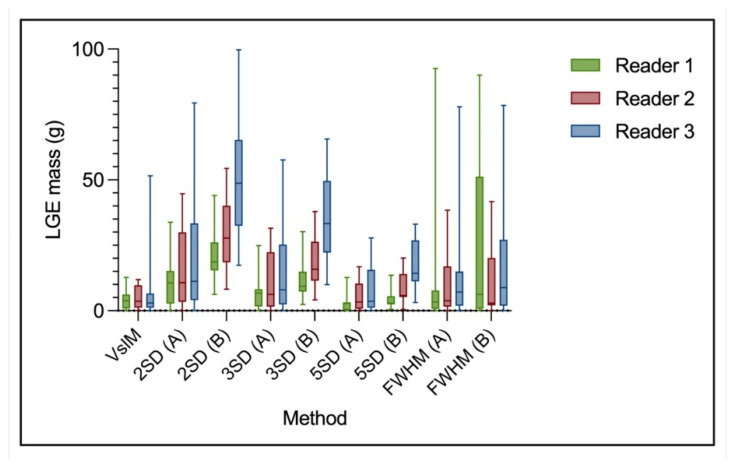
Column graph comparing post-processing method (A) vs. (B) for the STRM and FWHM techniques. In STRM method A, a normal myocardial ROI was drawn only in slices containing LGE compared to a normal myocardial ROI drawn in every single slice for method B. Using the VslM as the reference, method B consistently produced higher absolute LGE masses compared to method A across all standard deviation thresholds. Similarly, method B (multiple ROIs) for the FWHM technique yielded higher absolute values compared to method A (a single ROI), but these differences were smaller than for the STRM methods. LGE—late gadolinium enhancement, VslM—visual slider method, 2SD (A)—two standard deviations method A, 2SD (B)—two standard deviations method B, 3SD (A)—three standard deviations method A, 3SD (B)—three standard deviations B, 5SD (A)—five standard deviations method A, 5SD (B)—five standard deviations method B, FWHM (A)–full width at half maximum method A, FWHM (B)—full width at half maximum method B, ROI—region of interest.

Two different post-processing methods were tested for the 2SD, 3SD, 5SD and FWHM techniques. For the STRM method, myocardial ROIs drawn in every slice of the short-axis stack (method B) consistently yielded higher LGE masses compared to method A across all three standard deviation thresholds and for all three readers (Figure 3). Similarly, method B for FWHM, which utilised multiple scar ROIs rather than a single scar, also yielded higher LGE masses for all three readers. The magnitude of the absolute differences for FWHM was, however, small (Supplementary Table S3). Bias for all semi-automated methods (2SD, 3SD, 5SD, FWHM) was higher using method B compared to method A (Supplementary Table S2).

### 3.4. Inter-Reader Reliability/Precision

The ICCs evaluating reliability amongst the three readers for each method are shown in Table 2. The ICC for VslM was acceptable at 0.7 (*p* < 0.001). The 2SD (A) method yielded the best reliability (ICC = 0.9 with *p* < 0.001) while the fully automated OAT method yielded unacceptable reliability (ICC = 0.5, *p* < 0.001). Both the 3SD (A) and FWHM (A) methods yielded clinically acceptable reliability (ICCs = 0.8 and 0.8, respectively). Amongst the (B) methods, the FWHM (B) method yielded a good ICC of 0.8 (*p* < 0.001), but the remainder of the (B) methods yielded poor reliability results (ICCs < 0.4 with *p* values < 0.001) (Supplementary Table S4).

## 4. Discussion

This study, comparing different LGE quantification techniques in the context of AS, showed that the choice of method (VslM/STRM/FWHM/OAT) and post-processing style significantly influenced the accuracy and reproducibility of the LGE burden as determined by three independent CMR readers.

The most accurate methods emerging from this study included the 5SD STRM and FWHM methods. These findings are consistent with data from other studies that have performed a head-to-head analysis of different LGE quantification techniques in non-ischaemic cardiovascular diseases (hypertrophic and dilated cardiomyopathy) [29,31,32]. The scarring in these non-ischaemic pathologies share similarities with AS, which include the presence of relatively non-bright scars with concomitant diffuse interstitial fibrosis [29,31,32]. In two of these studies [29,31], a visual method was also used as a reference. The 5SD STRM, 6SD STRM and FWHM methods were found to be most accurate [29,31]. In the third study, histology from whole explanted hearts was used as a reference, and the 6SD STRM method emerged as most accurate, while the FWHM technique was not evaluated [32]. The finding that higher thresholds (5 or 6SDs) were more accurate than 2 or 3SD thresholds likely reflects the impact of high background diffuse interstitial fibrosis, which broadens the range of signal intensities across the myocardium. The STRM method is based on standard deviations. Therefore, a broader range of signal intensities increases the range of pixels that would be considered as enhanced, and to avoid scar overestimation, a higher standard deviation threshold is required. This challenge also exists in AS, where replacement fibrosis often coexists with diffuse interstitial fibrosis and plausibly explains our findings, where the high standard deviation thresholds were found to be more accurate compared to the lower (2–3 SDs) thresholds.

In the context of AS specifically, a direct comparison of different LGE quantification techniques remains exceedingly rare. Research that compared different STRM thresholds against histology found the 3SD STRM method to be most accurate [8]. The difference in the accuracy results between their study and ours may have resulted from the different reference methods used (histology vs. the VslM in our study). The histological examination by Treibel et al. described the presence of microscars measuring smaller than the typical voxel size on CMR [8]. Since each voxel represents an averaged signal intensity, voxels occupied by both normal myocardial tissue and one or more microscars may reflect a relatively lower signal intensity than expected of scar. The implication of this is that microscars on CMR are potentially missed by both the eye of the reader (visual method) and a high standard-deviation threshold (e.g., 5SD) [21,22,28]. This may explain the high accuracy of the 3SD method when histology is used as the reference (where microscars are more easily diagnosed), compared with the better accuracy of the 5SD method in our study, which used a visually guided estimate as the reference.

In our study, accuracy was assessed by comparing median LGE quantities for each semi-automated method to those of the VslM assessment rather than histology, which is considered the gold standard for fibrosis evaluation [19,20]. Although there is no consensus recommendation on which LGE quantification technique is optimal for non-ischaemic pathologies, including AS, the guideline recommendation for LGE quantification in general emphasises the initial use of a visual assessment, even when a semi-automated approach is used [24]. Thus, in clinical practice, an informed evaluation is usually made using the information from both quantification strategies. The traditional visual method requires the CMR reader to estimate scar burden according to the cumulative transmural LGE extent in each of the seventeen AHA segments [24]. This visual assessment is, by its nature, subjective. The visual assessment performed in this study (the VslM approach), however, avoided the need for a user-derived estimate of transmural extent (thereby limiting some of the subjectivity/bias) by use of a slider function in the post-processing software. This function provided an automated LGE mass, determined quantitatively by summing the pixels in a manually defined region of scar (Figure 1), guided by the visual assessment of the reader. Although the semi-automated methods were developed with the aim of improving objectivity, they still rely on visual input from the user who manually adjusts contours and ROIs until satisfied with the visual feedback from the software, which marks/highlights the areas it has counted as scar. For these reasons, we considered the VslM a reasonable reference in the absence of histology for comparing the accuracy of the semi-automated methods.

While accuracy is an important factor to consider when agreeing on an LGE quantification method, reproducibility is of equal importance if we are to adopt LGE quantification into widespread clinical practice. The 2SD STRM method had the best inter-reader reproducibility in this study. However, a low threshold masks the more subtle impact of inherent signal heterogeneity on the final outcome. This allows for better inter-reader reproducibility but at the expense of significant overestimation of the scar burden, as shown by the clinically unacceptable accuracy of the 2SD STRM method in our data. Good reproducibility was also shown for the 3SD STRM and FWHM methods. In the case of the FWHM method, it has often presented itself as one of the most reproducible techniques in other non-ischaemic pathologies, including hypertrophic cardiomyopathy [29,31]. This is in part related to the fact that the ROI is drawn in a region of scar, therefore negating the challenge of drawing a normal myocardial ROI in myocardium that is also burdened with diffuse interstitial fibrosis. The combination of its good reproducibility and statistically acceptable accuracy makes the FWHM method a potentially well-balanced candidate for scar quantification in AS, but it is not without limitations.

Although the means of the LGE mass differences between the FWHM and VslM methods for all three readers were small (<1 g), the ranges of the mass differences were wide (Figure 2). On closer inspection, large discrepancies in median values between the FWHM and VslM methods mostly arose in cases where scars were small, non-bright, or both. In our cohort of AS, scar has been found to be heterogeneous from patient to patient. While non-ischaemic bright-signal-intensity scars that are suitable for quantification by the FWHM method (which assumes a bright core) [25,28] are seen, several patients also present with small, non-bright scars. A single unifying semi- or fully automated method that accounts for this heterogeneity in the nature of the scarring while remaining both accurate and precise may not realistically exist.

Use of the semi- and fully automated methods was expected to limit the subjectivity associated with the visual method. These methods, however, still require significant user input as mentioned. To illustrate, the STRM and FWHM methods require the user to start with the visual identification of the scar. The ROIs drawn are also user-guided, and in this cohort, the differences in the ROIs drawn contributed to some of the wide variation observed using the semiautomated methods. For example, when readers visually estimated an LGE mass within 1 g of each other, the 2SD STRM method in the same patients yielded LGE mass differences that were nearly double one another. In these cases, systematic bias alone could not explain these differences, as proportional differences would have been expected. The variation, therefore, must have arisen from differences in the location and/or size of the ROI. The three readers in this study were selected from different institutions with different levels of education and experience with regard to CMR. Despite this, inter-reader reproducibility for the VslM estimations was statistically acceptable. An argument can be made that, if a single unifying semi-automated method in the context of AS does not exist, the visual method, when performed in the manner described in this article (VslM), may be sufficient for scar quantification in AS.

The impact of post-processing style has previously been speculated to impact the accuracy of LGE quantification, yet this remains to be systematically investigated in the existing AS literature. In alignment with prior speculation, our data showed that the style of post-processing impacted both the accuracy and reproducibility of LGE quantification in AS. The STRM (B) method, which utilised a myocardial ROI in every slice, including those without visible LGE, was the least accurate and least reproducible across standard deviation thresholds. The STRM (B) method consistently overestimated scar burden relative to the VslM. This was expected since every single slice would have yielded an enhanced proportion by the mathematical nature of the technique at the range of thresholds tested (the magnitude of the threshold is determined by the standard deviation of pixels within the ROI—therefore, for as long as there is a ROI, there will always be a standard deviation and unless an extremely high threshold is selected, there will always be a proportion of pixels in the rest of the slice that exceeds the chosen threshold). One argument is that we should include a myocardial ROI on every slice (including those without visible LGE) to be truly objective in quantifying LGE with the STRM method. The argument here is that the software is objectively identifying pixels with intermediate signal intensity that the eye of the CMR reader would otherwise ignore and is therefore offering a less biassed estimate. Though this is only true if the studies that validated a given threshold against histology analysed their LGE in this manner (i.e., using the STRM B method). Unfortunately, histological validation studies of the various methods are already rare, and this vital information is rarely reported on.

In real-world practice, AS often coexists with CAD. Myocardial infarction from obstructive CAD manifests in the form of subendocardial LGE, and evidence suggests that infarct scar in AS carries a similarly elevated risk of mortality as non-infarct scar [11]. In our cohort, patients with significant CAD and prior myocardial infarction were excluded. Therefore, the impact of the infarct scar on the results of this study is unknown. This is important given that the infarct scar is often bright on LGE, in contrast with the relatively non-bright, heterogeneous signal-intensity scarring observed in AS. Previous studies, including the ReLate Study, which analysed one of the largest cohorts in the literature, showed that the FWHM technique was the most reproducible for the quantification of infarction-associated LGE [31,33]. The performance of manual thresholding, on the other hand, was least reproducible [31,33]. This highlights the heterogeneity of myocardial scarring in cardiovascular disease and illustrates the complexity of recommending a single LGE quantification technique that uniformly accounts for this heterogeneity in clinical practice, where dual pathology may be encountered.

## 5. Limitations

A histological analysis was not performed in this study, therefore limiting the ability to test accuracy against what is considered the gold standard. It must be noted, however, that endomyocardial tissue is typically sampled from the basal septum. This represents one of seventeen LV myocardial segments, compared with CMR, which visualises the entire ventricle. This is an important consideration given strong evidence that a myocardial fibrosis gradient exists in AS. In our own cohort, septal predominance in fibrosis burden was also observed, in addition to the basal-to-apical gradient [34]. Therefore, while endomyocardial biopsy is considered the gold standard for fibrosis detection and quantification, it is not without relevant limitations, including the lack of complete representation of the LV.

An inter-reader intraclass correlation analysis based on myocardial regions and/or myocardial segments was not performed in this study. Given the observation of a fibrosis gradient in patients with severe AS, this analysis may offer further insights in future studies.

## 6. Conclusions

Scar quantification may serve as a useful parameter for risk stratification of high-risk AS patients who do not meet current guideline recommendations for valve intervention. However, no consensus exists on which quantification method is optimal for this population of patients. This study compared various scar quantification techniques in the context of severe AS. In addition to previously published studies on scar quantification in AS, it also included the FWHM and OAT methods, as well as an additional analysis on how post-processing styles may impact the accuracy and reproducibility of LGE quantification. We found that the visual method, when performed in the manner described in this article (i.e., the VslM approach), may be reasonably sufficient. Where a semi-automated method is preferred, the FWHM method utilising a single scar without ROI extrapolation appears to offer a reasonable balance in terms of accuracy and reproducibility. Importantly, the heterogeneous nature of myocardial scarring in AS may limit the use of the FWHM method in some cases, including those with small or non-bright scars. Larger studies designed with histological or clinical outcome-linked validation are still needed.

## Figures and Tables

**Table 1:** Baseline characteristics of the study population.

Parameter	Population (n = 15)
Age (years)	61 ± 14
Sex	
Female *n* (%)	12 (80)
Male *n* (%)	3 (20)
Hypertension *n* (%)	13 (87)
Diabetes Mellitus *n* (%)	5 (33)
Coronary artery disease *n* (%)	0 (0)
AVA (cm^2^)	0.51 ± 0.15
Mean gradient (mmHg)	61 ± 18
Peak gradient (mmHg)	96 ± 28
LVEF (%)	47 ± 18
SBP (mmHg)	126 ± 22
DBP (mmHg)	73 ± 12

Continuous variables are presented as mean ± standard deviation. Categorical variables are presented as absolute numbers (percentages). AVA—aortic valve area, LVEF—left ventricular ejection fraction, SBP—systolic blood pressure, DBP—diastolic blood pressure.

**Table 2:** Intraclass correlation coefficients for inter-reader reliability using a two-way random effects model.

	VslM	2SD (A)	3SD (A)	5SD (A)	FWHM (A)	OAT
ICC	0.7	0.9	0.8	0.6	0.8	0.5
*p* value	<0.001	<0.001	<0.001	<0.001	<0.001	<0.001

A *p*-value < 0.05 is considered statistically significant. ICC—intraclass correlation coefficient, VslM—visual slider method, 2SD (A)—two standard deviations method A, 3SD (A)—three standard deviations method A, 5SD (A)—five standard deviations method A, FWHM (A)—full width at half maximum method A, OAT—Otsu auto threshold.

## Data Availability

The dataset analysed for this publication is available upon reasonable request to the corresponding author.

## Supplementary Materials

The following supporting information can be downloaded at: https://www.mdpi.com/article/10.3390/diagnostics16132017/s1, Table S1: Wilcoxon rank-sum tests and Spearman rho correlation analyses; Table S2: Bland-Altman Analyses; Table S3: Wilcoxon rank-sum tests and Spearman correlation analyses for the B methods; Table S4: Intraclass correlation coefficient analyses for the B methods.

## Abbreviations

AS	aortic stenosis
SAVR	surgical aortic valve replacement
TAVI	transcatheter aortic valve implantation
CMR	cardiovascular magnetic resonance
LGE	late gadolinium enhancement
LV	left ventricle/left ventricular
VslM	visual slider method
SDs	standard deviations
STRM	signal threshold versus reference mean method
FWHM	full-width at half-maximum method
OAT	Otsu auto threshold method
CAD	coronary artery disease
TI	inversion time
TE	time to echo
TR	time to repeat
ROI	region of interest
ICC	intraclass correlation coefficient
