# Supplementary material for: Analysis of the Accuracy and Inter-Reader Precision of Scar Quantification Techniques in Aortic Stenosis: A Comparative Cardiovascular Magnetic Resonance Imaging Study
Source: Diagnostics (Basel). 2026 Jun 28;16(13):2017. doi: 10.3390/diagnostics16132017 (PMC13360222; doi:10.3390/diagnostics16132017)
Supplement: Supplementary file 1 [file diagnostics-16-02017-s001.zip › diagnostics-4147190-supplementary.pdf]

Analysis of the Accuracy and Inter-Reader Precision of Scar Quantification Techniques in Aortic Stenosis: A Comparative Cardiovascular Magnetic Resonance Imaging Study

Megan Rian Rajah [1,*], Pieter-Paul Strauss Robbertse [1], Vishesh Sood [2], Tonya Marianne Esterhuizen [3], Anton Frans Doubell [1] and Philip George Herbst [1]

1 Division of Cardiology, Department of Medicine, Faculty of Medicine and Health Sciences, Stellenbosch University, Tygerberg Hospital, Cape Town 7505, South Africa
2 Department of Radiology, SCP Radiology, Panorama Mediclinic Hospital, Cape Town 7500, South Africa
3 Division of Epidemiology and Biostatistics, Department of Global Health, Stellenbosch University, Cape Town 7505, South Africa
* Correspondence: 16607031@sun.ac.za

Supplementary Section

Supplementary Table S1. Wilcoxon rank-sum tests and Spearman rho correlation analyses.

	2SD (A)		3SD (A)		5SD (A)		FWHM (A)		OAT	
	Mean of differences	***p value***	***Mean of differences***	***p value***	***Mean of differences***	***p value***	***Mean of differences***	***p value***	***Mean of differences***	***p value***
Reader 1	1.17	0.001	0.75	0.03	-0.03	0.04	0.69	0.94	2.81	< 0.0001
Reader 2	0.86	0.002	0.53	0.01	0.03	0.52	0.30	0.15	2.20	< 0.0001
Reader 3	1.12	0.001	0.85	0.001	0.32	0.27	0.56	0.01	2.77	< 0.0001
	Spearman Rho	***p value***	***Spearman Rho***	***p value***	***Spearman Rho***	***p value***	***Spearman Rho***	***p value***	***Spearman Rho***	***p value***
Reader 1	0.9	0.0001	0.9	< 0.0001	0.8	0.002	0.8	0.001	0.4	0.2
Reader 2	0.9	< 0.0001	0.9	0.0001	0.8	0.0003	0.9	< 0.0001	0.2	0.4
Reader 3	0.8	0.0003	0.8	0.0003	0.9	0.0001	0.8	0.0008	0.1	0.8

Supplementary Table S2. Bland-Altman Analyses

	2SD (A)		3SD (A)		5SD (A)		FWHM (A)		OAT	
	Bias	***SD***	***Bias***	***SD***	***Bias***	***SD***	***Bias***	***SD***	***Bias***	***SD***
Reader 1	1.17	0.64	0.75	0.84	-0.03	1.37	0.69	1.26	2.81	1.60
Reader 2	0.86	0.82	0.53	0.79	0.03	0.76	0.30	0.63	2.20	0.94
Reader 3	1.12	0.87	0.85	0.78	0.32	0.69	0.56	0.85	2.77	1.08
	**2SD (B)**		**3SD (B)**		**5SD (B)**		**FWHM (B)**			
	Bias	***SD***	***Bias***	***SD***	***Bias***	***SD***	***Bias***	***SD***		
Reader 1	2.21	1.30	1.56	1.22	0.39	1.11	1.34	1.12		
Reader 2	1.99	0.59	1.48	0.53	0.49	0.68	0.45	0.69		
Reader 3	2.64	0.97	2.24	0.93	1.49	0.89	0.80	0.94		

Supplementary Table S3. Wilcoxon rank-sum tests and Spearman correlation analyses for the B methods.

	**2SD (B)**		**3SD (B)**		**5SD (B)**		**FWHM (B)**	
	Mean of differences	***p value***	***Mean of differences***	***p value***	***Mean of differences***	***p value***	***Mean of differences***	***p value***
Reader 1	2.21	< 0.0001	1.56	< 0.0001	0.39	0.99	1.34	0.02
Reader 2	1.99	< 0.0001	1.47	< 0.0001	0.49	0.002	0.45	0.01
Reader 3	2.64	< 0.0001	2.24	< 0.0001	1.48	0.005	0.80	0.003
	Spearman Rho	***p value***	***Spearman Rho***	***p value***	***Spearman Rho***	***p value***	***Spearman Rho***	***p value***
Reader 1	0.8	0.0002	0.9	< 0.0001	0.7	0.002	0.7	0.003
Reader 2	0.9	0.0001	0.8	0.0002	0.8	0.0007	0.8	0.0004
Reader 3	0.4	0.2	0.4	0.1	0.5	0.04	0.8	0.0009

Supplementary Table S4. Intraclass correlation coefficient analyses for the B methods.

	2SD (B)	3SD (B)	5SD (B)	FWHM (B)
**ICC**	0.37	0.36	0.36	0.82
**p value**	< 0.001	< 0.001	< 0.001	< 0.001
